# Effectiveness of the Early Adolescent Skills for Emotions (EASE) Intervention in Alleviating Post-Traumatic Stress Disorder: A Systematic Review and Meta-Analysis of Randomized Controlled Trials

**DOI:** 10.1192/j.eurpsy.2025.378

**Published:** 2025-08-26

**Authors:** V. Astori, B. Westphalen Pomianoski, D. Lopes Vieira, M. Prätzel Ellwanger, M. Frizzo Messinger

**Affiliations:** 1 Escola Superior de Ciências da Santa Casa de Misericórdia de Vitória, Vitória; 2 Universidade Nove de Julho, São Paulo; 3 Universidade Federal de Minas Gerais, Belo Horizonte; 4 Universidade do Contestado, Mafra; 5 Universidade Federal do Rio Grande do Sul, Porto Alegre, Brazil

## Abstract

**Introduction:**

The Lancet Commission noted severe mental health service disparities in low- and middle-income countries facing crises. Regarding this, the World Health Organization (WHO) developed the early adolescent skills for emotions (EASE), an experimental group-based intervention delivered by non-specialists, to offer evidence-based psychological support for adolescents with mental distress.

**Objectives:**

This systematic review and meta-analysis assesses EASE’s effectiveness in alleviating post-traumatic stress disorder (PTSD) compared with enhanced treatment as usual (ETAU).

**Methods:**

We systematically searched PubMed, Cochrane, and Scopus for randomized controlled trials (RCTs) comparing EASE with ETAU. Our outcomes included the overall improvement in post-traumatic and depressive symptoms, measured by the Children’s Revised Impact of Event Scale (CRIES-13) and the Patient Health Questionnaire-Adolescent version (PHQ-A) scores respectively. We pooled mean differences (MD) with 95% confidence intervals (CI) in RStudio using a random-effects model. Heterogeneity was assessed with I² statistics.

**Results:**

Six RCTs were included with 1,417 patients, of whom 642 (45.3%) received EASE treatment. In 67% of the studies, Syria was the main location of RCT’s. A total of 688(49%) were female. CRIES-13 (MD = -0.18, 95% CI [-1.88, 1.52], p = 0.84, I² = 0%; Figure 1) and PHQ-A (MD = -0.69, 95% CI [-1.47, 0.09], p = 0.08, I² = 15%; Figure 2) scores in the EASE intervention group compared to the ETAU group showed no significant difference. In both outcomes the heterogeneity was low.

**Image 1:**

**Figure:**
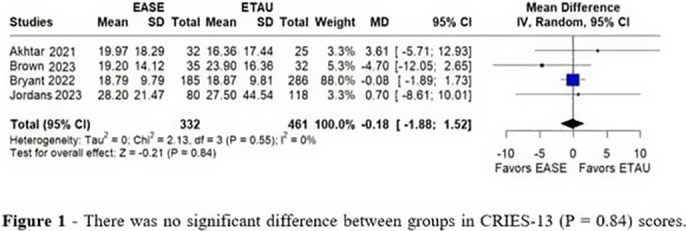

**Image 2:**

**Figure:**
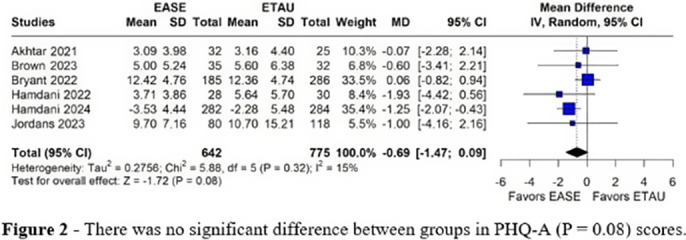

**Conclusions:**

The benefit of EASE in improving mental distress in adolescents is uncertain. Additional trials will provide new evidence for EASE potential therapeutic benefits.

**Disclosure of Interest:**

None Declared

